# Isolation and Characterization Cellulose Nanosphere from Different Agricultural By-Products

**DOI:** 10.3390/polym14132534

**Published:** 2022-06-21

**Authors:** Orapan Romruen, Pimonpan Kaewprachu, Thomas Karbowiak, Saroat Rawdkuen

**Affiliations:** 1 Food Science and Technology Program, School of Agro-Industry, Mae Fah Luang University, Chiang Rai 57100, Thailand; orapan.rom13@lamduan.mfu.ac.th; 2 College of Maritime Studies and Management, Chiang Mai University, Samut Sakhon 74000, Thailand; pimonpan.k@cmu.ac.th; 3 Cluster of Innovative Food and Agro-Industry, Chiang Mai University, Chiang Mai 50100, Thailand; 4 UMR PAM-Food and Wine Science & Technology, Agro-Sup Dijon, Université de Bourgogne France Comte, Esplanade Erasme, F-21000 Dijon, France; thomas.karbowiak@agrosupdijon.fr; 5 Unit of Innovative Food Packaging and Biomaterials, School of Agro-Industry, Mae Fah Luang University, Chiang Rai 57100, Thailand

**Keywords:** acid hydrolysis, agricultural waste valorization, cellulose nanosphere, functional property, homogenization, ultrasonication

## Abstract

Cellulose nanospheres (CN) have been considered a leading type of nanomaterial that can be applied as a strengthening material in the production of nanocomposites. This work aimed to isolate and characterize the properties of CN from different agricultural by-products. CNs were successfully isolated from rice straw, corncob, Phulae pineapple leaf and peel using acid hydrolysis (60% H_2_SO_4_) combined with homogenization-sonication (homogenized at 12,000 rpm for 6 min and ultrasonicated for 10 min). The results showed that the CN from rice straw (RS-CN) and corncob (CC-CN) exhibited high yields (22.27 and 22.36%) (*p* < 0.05). All hydrolyzed CNs exhibited a spherical shape with a diameter range of 2 to 127 nm. After acid hydrolysis, Fourier transform infrared (FTIR) results showed no impurities. X-ray diffraction (XRD) showed that the structure of cellulose was changed from cellulose-I to cellulose-II. However, cellulose-I remained in pineapple peel cellulose nanosphere (PP-CN). The crystalline index (CI) ranged from 43.98 to 73.58%, with the highest CI obtained in the CC-CN. The CN from all sources presented excellent thermal stability (above 300 °C). The functional properties, including water absorption Index (WAI), water solubility index (WSI) and swelling capacity were investigated. PP-CN showed the highest WAI and swelling capacity, while the PL-CN had the highest WSI (*p* < 0.05). Among all samples, CC-CN showed the highest extraction yield, small particle size, high CI, and desirable functional properties to be used as a material for bio-nanocomposites film.

## 1. Introduction

Petroleum-based polymers are non-biodegradable, with harmful pollutants discharged into the atmosphere during their manufacturing, recycling, and disposal [1]. Due to the depletion of fossil fuels and increased environmental concerns, the development and use of green biomass-based materials obtained from renewable resources have been intensively studied worldwide [2]. Biomass utilization has witnessed growing attention as a promising alternative to fossil fuels. Biomass waste comprises forest residues, agricultural leftovers, agro-industrial waste, animal waste, and urban waste. Agricultural residues are part of the plant that are ordinary discarded i.e., crop stalks, leaves, roots, fruit peels and seeds [1]. Large amounts of agricultural waste biomasses are produced and used globally. Thailand is an agricultural country; thus, large amounts of agricultural waste will be left over after harvesting. Rice straw, corncob, Phulae pineapple leaves, and peel were the main agricultural byproducts of Chiang Rai, Thailand. Unsuitable waste management of the materials mentioned above affected the environment. Therefore, the utilization of these byproducts as cellulosic products could solve the waste problem and is a way to add value addition to these wastes.

Agricultural residues are predominantly composed of cellulose, lignin, hemicellulose, pectin, and other minor chemicals [3]. Cellulose is considered a long-term sustainable alternative to synthetic polymers and has been used as a major raw material in several industries. It is the most prevalent renewable polymer, and due to its low costs, sustainability, biocompatibility, and biodegradability, it has become a crucial element of sustainable development [4]. Cellulose can be synthesized into a smaller structural material described as nanocelluloses using various techniques, such as enzymatic treatment and mechano-chemical techniques [5]. Nanocellulose is considered a leading type of nanomaterial that can be applied as a strengthening material in the production of nanocomposites because of its distinctive qualities, including biodegradability, renewability, non-toxicity, high mechanical strength, and optical properties [6,7]. Cellulose nanosphere (CN) is a novel type of nanocellulose with a spherical shape and size in the nanometer range [4]. The shape of nanoparticles significantly affects their properties and applications [8]. CNs are well accepted in a wide range of applications, such as drug delivery, disease detection, and diagnosis in the biomedical field [9].

The isolation methods of nanocellulose material commonly used are mechanical [10], chemical [11,12], chemo-mechanical [13], and a combination of all of them. Nanocellulose is typically produced by strong acid hydrolysis to remove amorphous components and to obtain a high crystalline index [14]. The quality of nanocellulose materials is affected by the source of cellulose, pretreatment, isolation method, and isolation conditions [15]. Recently, ultrasonication has been applied in many studies during post-treatment to break down CN aggregates in suspension via cavitation of ultrasonic waves [4]. For the production of nanocellulose, a combination of homogenization and ultrasonication is used as a non-hazardous, time-saving, and organic solvent-free process [16]. Ultrasonication and homogenization is an excellent method for the purification and production of smaller nanocellulose particles due to their strong power and convenience [17]. Hemmati, Jafari and Taheri [16], hydrolyze cotton cellulose to nanocellulose using 55–65% (*w*/*v*) H_2_SO_4_ for 3–7 min at various homogenization speeds, followed by sonication. The production yield of nanocellulose obtained was 59–72%, and the crystallinity index was 82%. The size of nanocellulose was 133 nm, with a 10 nm in width. However, the thermal stability of nanocellulose was less compared to raw cellulose.

Extraction and characterization of cellulose from different agricultural by-products (rice straw, corncob, pineapple leaf, and peel) were reported previously by Romruen, et al. [18]. Production of cellulose nanospheres (CN) from the aforementioned extracted cellulose could improve properties and increase the range of application. Therefore, this study aimed to hydrolyze CN from the by-products mentioned above using acid hydrolysis combined with homogenization and ultrasonication techniques. The properties of CNs including morphology, changes in chemical behaviors, crystallinity, thermal and functional properties have been investigated. All of the measured properties were compared with CN isolated from commercial cellulose.

## 2. Materials and Methods

### 2.1. Materials

Rice straw (RS) (*Oryza sativa* var. Glutinosa), corncob (CC) (*Zea mays* var. Indentata), Phulae pineapple (*Ananas comosus* var. Phulae) leaves (PL) and peels (PP) were collected from the agricultural fields of Thasud, Chiang Rai, Thailand. Commercial cellulose from cotton was purchased from Chanjao Longevity Co., Ltd. (Bangkok, Thailand). Sulfuric acid (H_2_SO_4_, 98%) was purchased from QRëC™ (Auckland, New Zealand). All the materials and chemicals used in our study were of analytical grade.

### 2.2. Cellulose Nanosphere Isolation

All agricultural by-products (rice straw, corncob, pineapple leave, and pineapple peel) were prepared and extracted according to the method described by Romruen, Karbowiak, Tongdeesoontorn, Shiekh and Rawdkuen [18] to obtain extracted cellulose samples.

Cellulose nanospheres (CN) were isolated using the modified method of Hemmati, Jafari and Taheri [16]. First, the extracted cellulose was hydrolyzed with 60% H_2_SO_4_ (1:20, *w*/*v*) and homogenized (T18 basic, Ika Labotechnik Janke & Kunkel Gmbh & Co., KG, Staufen, Germany) at 12,000 rpm for 6 min. After the hydrolysis, the mixture was centrifuged (MPW-352R, MPW Med. Instruments, Warsaw, Poland) at 10,000 rpm for 10 min (and repeated nine times). Next, the sediment was re-suspended in 100 mL of distilled water and homogenized for 2 min at 8000 rpm before being neutralized with 1M NaOH. The solution was then sonicated for 10 min (pulses of 2 s on and 1 s off) in an ice bath using an ultrasonic processor (Sonics & Materials, Inc., Model VCX 500, New Town, CT, USA). Finally, the solution was freeze-dried (Beta 2-8 LD plus, Martin Christ, Germany) for 24 h and stored in plastic containers for further study.

### 2.3. Yield of Cellulose Nanosphere

The extraction yield of CN was calculated from the proportion of the mass of the dried bagasse taken for cellulose nanosphere preparation following drying and the representative mass of bagasse using Equation (1).

(1) $$\mathrm{Yield}\;\left(\%\right)=\frac{\mathrm{weight}\;\mathrm{of}\;\mathrm{CN}\;\left(g\right)}{\mathrm{weight}\;\mathrm{of}\;\mathrm{drued}\;\mathrm{bagasse}\;\left(g\right)}\times 100\;$$

### 2.4. Scanning Electron Microscopy (SEM)

The morphologies of CN samples were observed under a field emission scanning electron microscope: FESEM (TESCAN, model, MIRA, Czech Republic). A droplet of the CN suspension (0.1%, *w*/*v*) was put on a stub, left to dry, sputtered with gold, and scanned at an accelerating voltage of 10 kV under 50 k× magnification.

### 2.5. Fourier Transform Infrared (FTIR) Analysis

FTIR spectra were determined at ambient conditions using an FTIR spectrophotometer (Perkin-Elmer/FTIR spectrum GX, Perkin-Elmer, Waltham, MA, USA) according to the conditions outlined by Lu and Hsieh [14]. Samples were ground with KBr (10 mg sample/1000 mg KBr) and pressed into transparent pellets for analysis. The spectra were obtained in transmittance mode from 32 scans with a resolution of 4 cm^−1^ over the 4000–400 cm^−1^ range. Baseline correction and normalization of the spectra were done using Perkin-Elmer software (Perkin-Elmer, Waltham, MA, USA).

### 2.6. X-ray Diffraction (XRD)

X-ray diffraction (XRD) patterns of CN were analyzed using an XRD diffractometer (X’Pert Pro MPD, PANalytical, Almelo, Netherlands) operated at 40 kV and 30 mA, equipped with Cu Kα radiation at a wavelength of 1.54056 Å and a nickel monochromator filtering wave. At room temperature, the samples were scanned over a temperature range of 2θ = 5 − 40° at a scanning rate of 0.4°/min. The percentage of crystallinity index (CI) was calculated following Equation (2):

(2) $$\mathrm{CI}\;\left(\%\right)=\frac{I_{c}}{I_{c}+I_{a}}\times 100$$

where I_c_ and I_a_ represent the integrated intensities of crystalline and amorphous regions, respectively.

### 2.7. Thermal Properties

The thermal stability (TGA & DSC) of CN was assessed through a thermogravimetric analyzer (TGA/DSC3+ HT, Mettler Toledo, Greifensee, Switzerland) following the conditions of Rashid and Dutta [19]. Approximately 5 mg of samples were subjected in an N_2_ gas atmosphere with a heating rate of 20 °C/min, the samples were heated from 25 °C to 600 °C, and the gas rate was 60 mL/min. DTG curves were used to calculate the onset (T_onset_) and maximum decomposition temperature (T_max_) of samples, while TGA curves were used to determine the char residue (%). DSC was used to calculate the onset (T_onset_), peak (T_max_), and enthalpy of crystallite melting (H, J/g).

### 2.8. Functional Properties of Cellulose Nanosphere

#### 2.8.1. Water Absorption Index (WAI) and Water Solubility Index (WSI)

The WAI and WSI of CN were established by the Kadan, et al. [20] methods of Kadan, et al. [20]. A one gram sample was suspended in 10 mL distilled water, then vortexed for 1 min. The mixture was heated at 30 °C in a water bath for 30 min with slight stirring, followed by centrifugation (MPW-352R, MPW Med. Instruments, Warsaw, Poland) at 3000 rpm for 10 min. After carefully pouring the supernatants into the moisture can, it was dried overnight at 105 °C to collect the dried supernatant. The following formulas were used to determine the WAI (Equation (3)) and WSI (Equation (4)):

(3) $$\mathrm{WAI}\;\left(g/g\right)=\frac{\mathrm{weight}\;\mathrm{of}\;\mathrm{wet}\;\mathrm{sediment}\;\left(g\right)}{\mathrm{dry}\;\mathrm{weight}\;\mathrm{of}\;\mathrm{CN}\;\left(g\right)}$$

(4) $$\mathrm{WSI}\;\left(\%\right)=\frac{\mathrm{weight}\;\mathrm{of}\;\mathrm{dried}\;\mathrm{supernatant}\;\left(g\right)}{\mathrm{dry}\;\mathrm{weight}\;\mathrm{of}\;\mathrm{CN}\;\left(g\right)}\times 100\;$$

#### 2.8.2. Swelling Capacity

The swelling capacity was determined by the method of Hassan, et al. [21]. The 0.2 g sample was precisely measured before moving it to a calibrated cylinder. The cylinder was then filled with 10 mL of distilled water. The mixture was then incubated at room temperature for 18 h. After that, the packed volume was measured, and swelling capacity was determined in mL/g of the sample following Equation (5):

(5) $$\mathrm{Swelling}\;\mathrm{capacity}\;\left(\mathrm{mL}/g\right)=\frac{\mathrm{water}\;\mathrm{displace}\;\left(\mathrm{mL}\right)}{\mathrm{dry}\;\mathrm{weight}\;\mathrm{of}\;\mathrm{CN}\;\left(g\right)}$$

### 2.9. Statistical Analysis

The data obtained in this study were analyzed with variance (ANOVA) analysis using statistical SPSS software (SPSS for Windows version 26, SPSS Inc., Chicago, IL, USA). The differences between means were performed using Duncan’s multiple range tests at a significance level of *p* < 0.05.

## 3. Results and Discussion

### 3.1. Morphology and Yield Analysis

**Figure 1:**
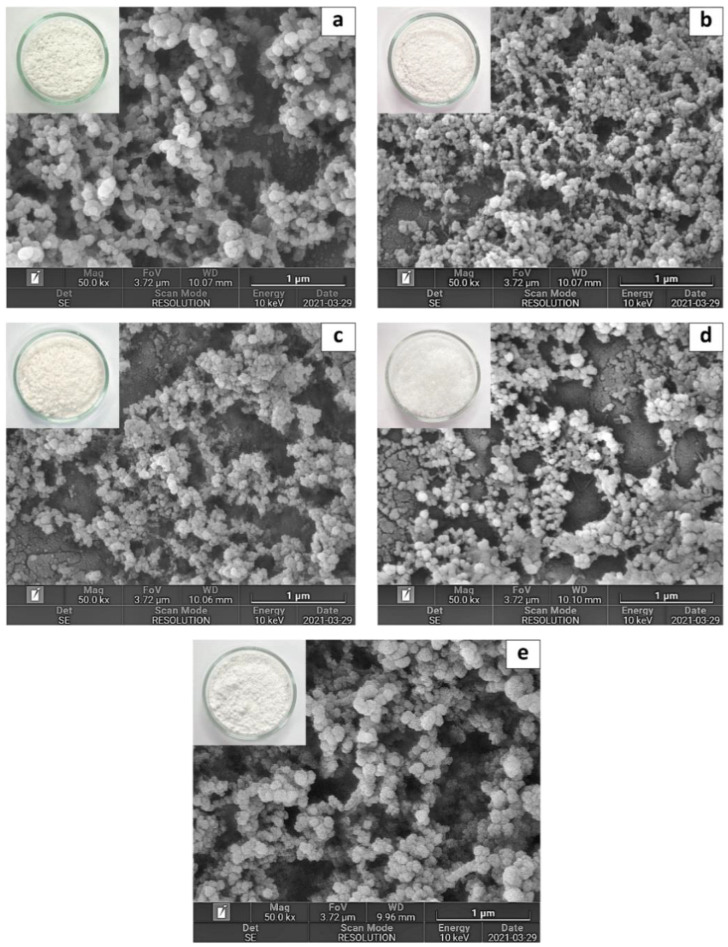
Scanning electron micrograph of cellulose nanosphere isolated from different sources compared with commercial cellulose: (**a**) rice straw, (**b**) corncob, (**c**) pineapple leaf, (**d**) pineapple peel, and (**e**) commercial cellulose (magnification 50 k×).

The cellulose of different agricultural by-products, including rice straw (RS), corncob (CC), Phulae pineapple leaf (PL), and peel (PP) were extracted and charactized their properties in our previous study (Romruen, Karbowiak, Tongdeesoontorn, Shiekh and Rawdkuen [18].The celluloses mentioned above were hydrolyzed into a nanoscale in this study. The cellulose nanosphere (CN) was isolated via acid (H_2_SO_4_) hydrolysis combined with homogenization and ultrasonication techniques. Commercial cellulose (COM), which uses the same process to hydrolyze the sample into nanoscale, was used to compare the properties. The extraction yield (calculated from the raw material weight) of RS-CN, CC-CN, PL-CN, and PP-CN was 22.27, 22.36, 12.06, and 6.69% (*w*/*w*), respectively (Table 1) (*p* < 0.05). Figure 1 displays the appearance and microstructural morphology of CN from various sources. The appearance of all samples presented with a white powder; white color is representative of cellulose. The FESEM results show that the morphology of extracted CN was consistently distributed as a spherical shape with a diameter range of 28–82, 2–44, 8–42, 11–36, and 28–127 nm for RS-CN, CC-CN, PL-CN, PP-CN, and COM-CN, respectively. Most CNs prepared by the acid hydrolysis process were reported at sizes below 100 nm [4].

The results are consistent with Mehanny, et al. [22]; nanocellulose obtained from palm wastes via 20% H_2_SO_4_ (*v/v*) at 120 °C for 30 min was reported in spherical particles of 42–82 nm diameter. However, the morphology of isolated CN was different from some previous studies, as they were rod-like or needle-shaped [11,23,24]. Yang, et al. [25], suspected that the resulting sphere shape of CN could be because of pretreatment processes such as milling, fiber regeneration prior to acid hydrolysis, and TEMPO-assisted oxidation. Alternatively, it could result from the ultrasonic treatment applied during the acid hydrolysis process. In the hydrolysis process, ultrasonication helps to enhance the contact between reactants, increasing cellulose hydroscopicity, facilitating acid molecular penetration, and accelerating the reaction rate [26]. The morphology of CN reveals some agglomeration due to the drying process, indicating that a strong, cohesive force exists inside the nanomaterials [27]. The cellulose nanosphere has a higher specific surface area and larger aspect ratios than nanocellulose with the same diameter [4]. Therefore, spherical shape nanocellulose has a stronger potential than rod-shaped nanocellulose to improve the crystallinity and thermal stability of a polymer matrix at the same nanocellulose concentration [28].

### 3.2. Fourier Transform Infrared (FTIR) Analysis

**Figure 2:**
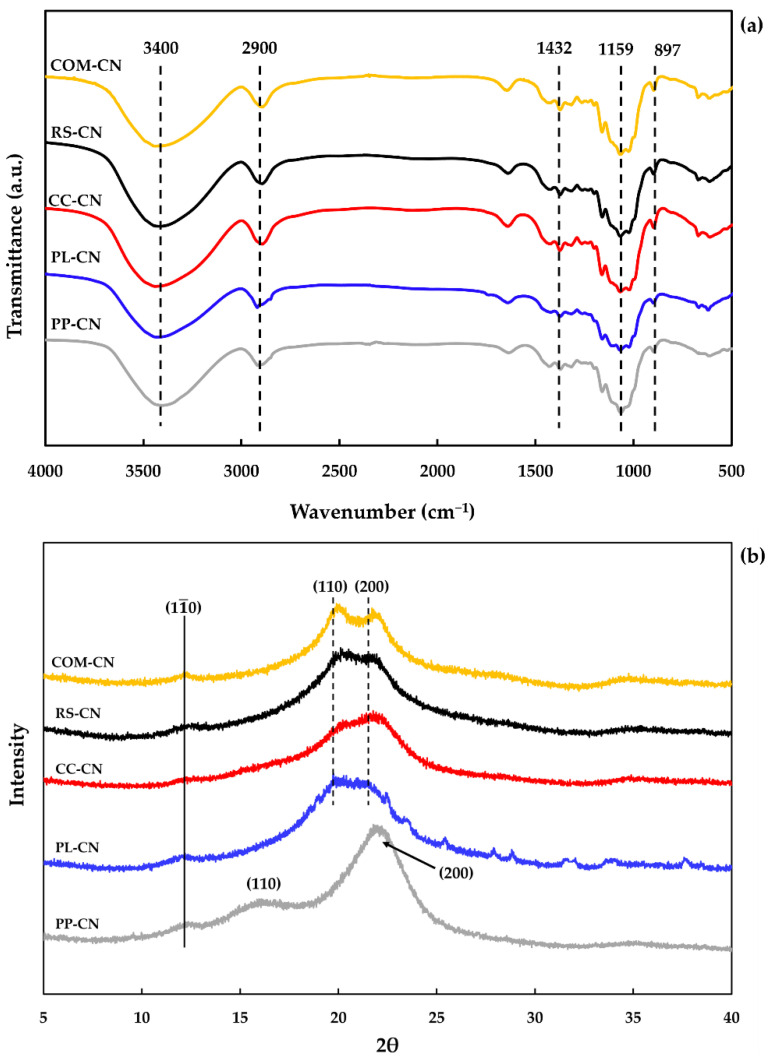
FTIR spectra (**a**) and XRD spectra (**b**) of cellulose nanosphere isolated from different sources compared with commercial cellulose. RS-CN: rice straw cellulose nanosphere; CC-CN: corncob cellulose nanosphere; PL-CN: pineapple leaves cellulose nanosphere; PP-CN: pineapple peels cellulose nanosphere; COM-CN: cellulose nanosphere hydrolyzed from commercial cellulose.

The chemical composition of the isolated CN was investigated using FTIR analysis, which identified the functional groups present. The FTIR spectra of extracted CN are presented in Figure 2a. The diffraction spectra of all samples were similar, demonstrating that the form of the raw material did not affect the chemical functionality of the isolated CN. A dominant band of 3450–3400 cm^−1^ and 2900 cm^−1^ contributed to the O-H and C-H stretching vibrations, corresponding to the aliphatic moieties in polysaccharides in all spectra [29]. Another spectrum observed at the wavenumber of 1640 cm^−1^ is related to the bending behavior of the O=H groups, affected by the absorption of water. This finding might well be in relation to water absorption due to the sulfate groups of sulfuric acid becoming placed on the surface after the hydrolysis process, improving the hydrophilic properties of CN [30]. The presence of the sulfate group has been confirmed by the absorption bands appearing around 1223–1234 cm^–1^ [31]. In addition, the peak of 1742 cm^−1^ corresponded to carboxylic acid and esters, which were a major component of the extracts observed in any of the samples [32]. It demonstrated that acid treatment completely hydrolyzed the hemicellulose traces. The absence of the peak at 1541 cm^−1^ (aromatic CC = benzene ring stretching) indicated the elimination of lignin residue. This is because of the acid hydrolysis process severely distroyed cellulose-lignin bonds [33]. Consequently, peak intensities at 897 cm^−1^, 1028 cm^−1^, 1106 cm^−1^, 1159 cm^−1^, and 1432 cm^−1^ accredit to the vibrations of cellulose bonds and rings became prominent after acid hydrolysis affect the increase in crystalline stability [19].

### 3.3. X-ray Diffraction (XRD) Analysis

The XRD spectra of isolated CN from different agricultural by-products are presented in Figure 2b, and the calculated crystallinity index (CI) is shown in Table 1. The diffraction peaks of RS-CN, CC-CN, PL-CN, and COM-CN have been observed at 12.1°, 20.0°, and 21.8°. The presented peak corresponds to crystallographic planes of ($1\bar{1}0$), (110), and (200), indicating the cellulose-II structure [34]. According to our previous study, all cellulose extracted from RS, CC, PL, and PP were presented in the cellulose I structure [18]. Therefore, these findings confirmed that the cellulose-II crystal structure was formed during the acid hydrolysis process. In contrast, PP-CN shows the peaks at 2θ around 12.1°, 16.0°, and 22.0°, respectively. The peak at 16.0° and 22.0° coincided with the characteristic diffraction maxima of cellulose-I, while the peak at 12.1° indicates that a minor quantity of cellulose-II is present [34]. The most common crystalline form in plants is cellulose-I, which comprises an assembly of crystallites and disordered amorphous areas [35]. Because cellulose-II has a more potent chemical reactivity than cellulose-I and can be converted into good cellophane, it is one of the most valuable fibers and has a wide range of applications in the chemical industry [36]. Cellulose-II can be obtained from cellulose-I via the regeneration and mercerization process [37]. CI assessed by XRD displays the changes in the physical and mechanical properties of the materials. The CI values of RC-CN, CC-CN, PL-CN, PP-CN, and COM-CN were 58.43, 73.58, 64.75, 43.98, and 63.23%, respectively (Table 1). The conversion of cellulose-I to cellulose-II was increased in the crystallinity index, which demonstrates the preferential destruction of cellulose’s amorphous regions during sulfuric acid hydrolysis [38]. However, the decrease in CI may be attributed to the loss of the crystallization region due to the breaking of hydrogen bonds among cellulose macromolecules [39].

### 3.4. Thermal Stability

**Figure 3:**
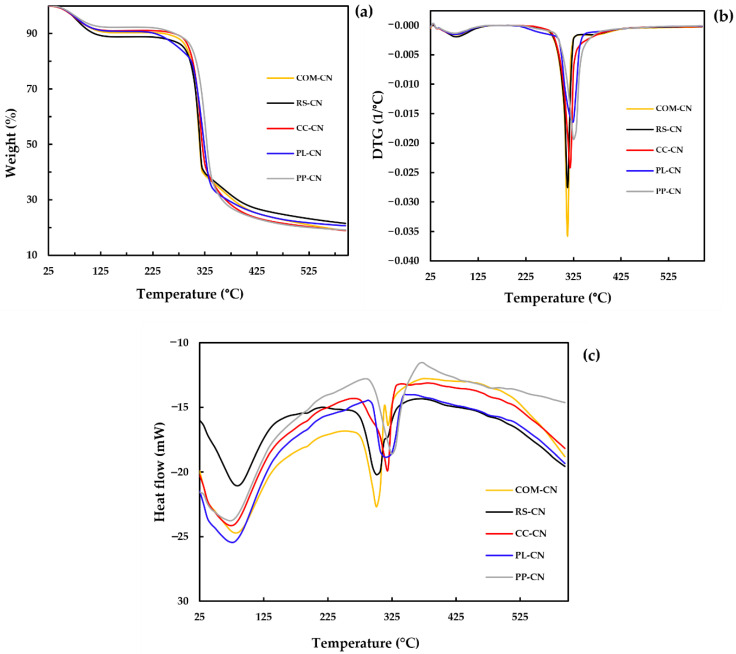
TGA (**a**), DTG (**b**), and DSC (**c**) curves of cellulose nanosphere isolated from different sources compared with commercial cellulose. RS-CN: rice straw cellulose nanosphere; CC-CN: corncob cellulose nanosphere; PL-CN: pineapple leaves cellulose nanosphere; PP-CN: pineapple peels cellulose nanosphere; COM-CN: cellulose nanosphere hydrolyzed from commercial cellulose.

In order to investigate the thermal characteristics of extracted CN, a TGA-DSC combination testing system was used. The evaluation of thermal stability is crucial for its possible application as a bio-nano composites reinforcing material. The thermal stability of polymer depends on the intrinsic properties of the samples and the molecular interactions between the various macromolecules [40]. The results of TGA thermograms, DTG, and DSC curves are shown in Figure 3, and the analysis results are summarized in Table 2. The initial weight loss of CN occurred around 60–125 °C, with 7–10% weight loss due to the evaporation of volatile chemicals and water [41]. The second degradation occurs at temperatures between 295 and 305 °C, mainly from hemicelluloses and lignin decomposition. The degradation temperatures (T_max_) of the isolated CN from RS, CC, PL, PP, and COM were found at 314, 318, 323, 327, and 313 °C, respectively. The small variance indicates that the CNS obtained are similar in composition and thermal stability. The final residues remain at 600 °C after thermal degradation, depending on the raw material type. The RS-CN shows the highest final residue (27.54%), followed by CC-CN (25.38%), COM-CN (25.29%), PL-CN (25.03%), and PP-CN (23.34%), respectively. The strong cellulose structure could endure high-temperature conditions, as shown by the comparatively low weight losses [42]. The residue of CNs after 600 °C was higher than their cellulose by a factor of 1.2–2.0 times [18]. Variations in chemical structure and crystallinity may influence the variances in final residues among the CNs. According to the acid hydrolysis process to produce CN, the sulfate ester groups worked as a flame retardant, which resulted in a high final residue of CN [43]. The low final residue of PP-CN could differ between cellulose crystal structure types. According to the results from XRD analysis, extracted CN was observed to be cellulose type-II except for PP-CN that presented in cellulose type I. The cellulose-II crystal structure was extremely thermally stable [44]. The thermal degradation behavior of extracted CN was consistent with the nanocellulose isolated from biomass [45,46].

The changing thermo-molecular behaviors of isolated CNs were evaluated with DSC curves, as shown in Figure 3c. The results demonstrate that all the samples had similar degradation patterns. In the beginning, an endothermic peak was observed at the temperature range of 30 to 130 °C, demonstrating that the absorbed heat energy was employed for water vaporization [47]. A small second endothermic peak occurred at 200 °C, showing the heat breakdown initiation of cellulose. An endothermic peak is observed at around 300 °C, corresponding to cellulose decarboxylation and depolymerization [48]. The extracted cellulose nanosphere’s onset degradation temperature (T_onset_) was 275.94–305.13 °C (Table 2). Additionally, the peak degradation temperatures (T_max_) were 302.00, 317.33, 318.00, 327.33, and 302.33 °C for RS-CN, CC-CN, PL-CN, PP-CN, and COM-CN, respectively. The degradation enthalpy (∆H) of extracted CN ranges from 120.44 to 191.44 J/g; the highest enthalpy was presented in PP-CN, while the lowest was in PL-CN. The resulting DSC values were remarkably similar to the TGA data, confirming that the DSC analysis was consistent with the TGA study.

### 3.5. Functional Properties

Functional properties of extracted CN, including water absorption index (WAI), water solubility index (WSI), and swelling capacity, are presented in Table 3. Several factors, including non-cellulosic fraction content, particle size, porosity, and crystallinity, can affect the functional properties of cellulosic materials [49,50]. The results show that the WAI, WSI, and swelling capacity of extracted and commercial CN were significantly different (*p* < 0.05). For WAI, PP-CN shows the highest value (14.24 g/g), followed by PL-CN, RS-CN, CC-CN, and COM-CN, respectively. For WSI, the significant highest value was presented in PL-NC, while the RS, PP, and COM-CN were not significantly different. The PPNC presented the highest swelling capacity (9.01 mL/g), followed by CC-CN, COM-CN, RS-CN, and PL-CN. Ultrasound waves generate gas bubbles to form and collapse, known as cavitation, which alters the surface structure of lignocellulosic materials, promoting porosity and increasing WAI, WSI, and swelling capacity [51]. A large number of hydroxyl groups in cellulose cause more hydrogen-bonding capacity, resulting in a higher ability to absorb water [52]. The size reduction from cellulose to nanocellulose produced a more uniform size and increased a specific surface area, resulting in a high water-holding ability [53]. For future applications in biopolymer film, higher WAI and WSI of cellulose nanospheres are needed to dissolve the cellulose nanosphere completely in the film formation process.

## 4. Conclusions

Cellulose nanospheres (CNs) were successfully isolated from four different agricultural by-products (rice straw (RS), corncob (CC), pineapple leaf (PL), and pineapple peel (PP) by acid hydrolysis combined with homogenization-sonication. The highest yields were presented in RS-CN (22.27%) and CC-CN (22.36%) (*p* *<* 0.05). The morphology results of CNs showed a spherical shape with a diameter range of 2 to 127 nm. FTIR results confirmed that no impurities remained after acid hydrolysis. XRD results indicated that the structure of cellulose was changed from cellulose-I to cellulose-II. However, cellulose-I remained in PP-CN. The CI of isolated CNs ranged from 43.98 to 73.58%, with the highest CI obtained in the CC-CN. The CNs from all sources presented excellent thermal stability (above 300 °C). The RS-CN shows the highest final residue (27.54%), followed by CC-CN (25.38%), COM-CN (25.29%), PL-CN (25.03%), and PP-CN (23.34%), respectively. In addition, the residue of CNs after 600 °C was higher than their cellulose by a factor of 1.2–2.0 times. All CNs exhibited superior thermostability, indicating that they could be applied as a viable bio-filler in the production of nanocomposite products. PP-CN showed the highest WAI and swelling capacity, while the PL-CN had the highest WSI (*p* < 0.05).

The present study established alternate solutions to produce cellulose nanospheres from undiscovered biomasses for several applications. In a future study, the cellulose nanosphere produced from the agricultural by-product will be used as a reinforcing material to improve the barrier properties in the biodegradable intelligent bilayer films. Moreover, the utilization of cellulose nanospheres from agricultural waste could solve the problem of the burning of agricultural wastes, which significantly increases particulate matter based on the amount of smoke generated, causing pollution and health concerns. On the other hand, it can be of added value to this waste.

## Figures and Tables

**Table 1:** Extraction yield, crystal phase and crystallinity index (CI) of cellulose nanosphere extracted from different sources compared with commercial cellulose.

Source of Cellulose Nanosphere	Extraction Yield (%, *w*/*w*) *	Crystal Phase	Crystallinity Index (%)
Rice straw	22.27 ± 0.46 ^a^	type II	58.43
Corncob	22.36 ± 0.14 ^a^	type II	73.58
Pineapple leave	12.06 ± 1.12 ^b^	type II	64.75
Pineapple peel	6.69 ± 0.54 ^c^	type I	43.98
Commercial cellulose	-	type II	63.23

* Values are presented as mean ± standard deviation (*n* = 3) and different superscripts (^a–c^) are significantly different (*p* < 0.05).

**Table 2:** Thermal properties of cellulose nanosphere from different sources compared with commercial cellulose.

Source of Cellulose Nanosphere	TGA Analysis			DSC Analysis		
	T_onset_ (°C)	T_max_ (C)	Residue at 600 °C (%)	T_onset_ (°C)	T_max_ (°C)	ΔH (J/g)
Rice straw	304.22	314.67	27.54	275.94	302.00	177.04
Corncob	301.60	318.00	25.38	305.13	317.33	133.55
Pineapple leaf	295.62	323.33	25.03	295.42	318.00	120.44
Pineapple peel	302.25	327.67	23.34	296.10	327.33	191.44
Commercial cellulose	305.49	313.67	25.29	276.24	302.33	176.14

TGA: thermo-gravimetric analyzer; DSC: differential scanning calorimeter; T_onset_: onset decomposition temperature; T_max_: peak decomposition temperature; ΔH: enthalpy change.

**Table 3:** Functional properties of cellulose nanosphere from different sources compared with commercial cellulose.

Source of Cellulose Nanosphere	WAI (g/g)	WSI (%)	Swelling Capacity (mL/g)
Rice straw	7.77 ± 0.15 ^c^	2.01 ± 0.09 ^c^	1.91 ± 0.07 ^c^
Corncob	6.82 ± 0.02 ^cd^	3.64 ± 0.22 ^b^	2.96 ± 0.03 ^b^
Pineapple leaf	9.30 ± 1.46 ^b^	9.57 ± 1.06 ^a^	1.89 ± 0.09 ^c^
Pineapple peel	14.24 ± 0.25 ^a^	1.57 ± 0.19 ^c^	9.01 ± 0.10 ^a^
Commercial cellulose	6.18 ± 0.41 ^d^	1.81 ± 0.20 ^c^	2.95 ± 0.03 ^b^

Values are presented as mean ± standard deviation (*n* = 3). Different superscripts (^a–d^) in each column are significantly different (*p* < 0.05). WAI: water absorption index, WSI: water solubility index.

## Data Availability

The data presented in this study are available on request from the corresponding author.
